# A critical role of toll-like receptor 2 (TLR2) and its’ *in vivo* ligands in radio-resistance

**DOI:** 10.1038/srep13004

**Published:** 2015-08-13

**Authors:** Fu Gao, Chaoxiong Zhang, Chuanfeng Zhou, Weimin Sun, Xin Liu, Pei Zhang, Jiaqi Han, Linfeng Xian, Dongchen Bai, Hu Liu, Ying Cheng, Bailong Li, Jianguo Cui, Jianming Cai, Cong Liu

**Affiliations:** 1 Department of Radiation Medicine, Faculty of Naval Medicine, Second Military Medical University, Shanghai 200433, PR China; 2 Department of Centre for Disease Prevention and Control, Chengdu Military Region, Chengdu 610021, China; 3 National Key Laboratory of Medical Immunology& Institute of Immunology, Second Military Medical University, Shanghai 200433, China; 4 Model Animal Research Center, Nanjing University, Nanjing, People’s Republic of China

## Abstract

The role of Toll-like receptor-2 (TLR2) in radio-resistance remained largely unknown. TLR2 knockout (TLR2^−/−^) mice received radiation of 6.5 Gy, and then were studied. We found that radiation resulted in more severe mortality and morbidity rates in TLR2^−/−^ mice. The cause of death in TLR2^−/−^ mice may be severe and persistent bone marrow cell loss. Injection of the TLR2 agonist Pam_3_CSK_4_ into wild type (WT) mice induced radio-resistance. Myd88^−/−^ mice were more susceptible to radiation. In conclusion, our data indicate that, similar to TLR4, TLR2 plays a critical role in radio-resistance.

Radiotherapy is one of the most commonly used anticancer therapies in the clinic^1^,^2^,^3^,^4^. However, the most serious adverse effect of ionizing radiation is the acute radiation syndromes especially the bone marrow cells (BMC) failure in the hematopoietic syndrome^5^,^6^. The extreme sensitivity of BMC to genotoxic stress is a key factor for the survival of the body. Although mechanisms of radiation injury have been elucidated and the adioprotectants for medical and biodefense applications have been, and continue to be developed^5^,^6^,^7^,^8^, knowledge in this area is still very limited.

Toll like receptors have a crucial role in the detection of microbial infection in mammals and insects^9^. To date, there are 13 types of TLRs with different functions in humans and mice^10^. In a previous study in 2008, *Burdelya et al.*^5^ indicated that a TLR5 agonist, CBLB502, showed radio-protective activity in mouse and monkey models by activation of the NF-κB pathway. Other studies also showed that the CBLB502 serves as a possible link between radio-resistance and the epithelial innate immune response^11^,^12^. In our previous study, we found that TLR4 played a critical role in radio-resistance^13^.

TLR2, TLR4 and TLR5 are in the same TLR family and they have many similarities in their signaling pathway^14^,^15^. TLR2 molecular expression is very broad and the function of TLR2 has been much studied^14^. Two studies demonstrated that TLR2 was involved in TBI sensitivity^16^,^17^, and Pam_3_CSK_4_ showed a significant *in vivo* radioprotective efficacy^16^. The signaling pathway involved in the reported TRL2- mediated radio-resistance remains unclear.

In this study, we confirmed that the radio-resistance effects of TLR2 by using TLR2^−/−^ mice. As Myd88 plays a central role in the innate and adaptive immune response^18^, and is the important adaptor of TLR2 pathways^14^. In this study, we confirmed the radio-resistance effects of TLR2, and indicated that Myd88 was necessary for radio-resistance mediated by TLR2.

## Material and Methods

### Mice and treatments

C57BL/6 mice, 5–6 weeks of old, and TLR 2 knock out mice were purchased from The Model Animal Research Center, Nanjing University. All mice were housed in a Specific Pathogen-Free (SPF) facility for all experiments. All animal experiments were performed in accordance with the National Institute of Health “Guide for the Care and Use of Laboratory Animals”^19^, with the approval of the Laboratory Animal Center of the Second Military Medical University, Shanghai. All efforts were made to minimize the number of animals used as well as any suffering.

### Reagents

Pam_3_CSK_4_ was obtained from EMC Microcollection (Tübingen, Germany)^20^. Flow cytometry staining buffer (eBioscience, San-Diego, CA, USA); Annexin V-FITC and propidium iodide (PI) double staining apoptosis assay kit were purchased from Bipec Biopharma (Cambridge, MA, USA). Anti-BrdU antibodies (Cell Signaling Technology, Cat. #5292) and rabbit anti-mouse IgG-horse radish peroxidase-conjugate antibodies were from Cell Signaling Technology; Ampicillin was from Changhai Hospital, (Shanghai, China). DMEM and RPMI 1640 medium and fetal calf serum were from PAA Laboratories, GmbH,(Pasching, Austria). BrdU and 2′7′-di-chlorofluorescein diacetate (DCFH-DA, Molecular Probes, Biyuntian, China).

### Cell culture

Cells were cultured in 6-well plates at 37 °C in a humidified atmosphere of 5% CO_2_ with DMEM and 1640 Medium (PAA, Austria) containing 10% fetal calf serum (PAA, Austria). Exponentially growing cells were used for experiments.

### Total-body irradiation

A ^60^Co irradiator was used for total-body ionizing irradiation. Un-anaesthetized mice were placed in well-ventilated plastic boxes and exposed to the ^60^Co-γ radiation at a distance of 3 m 4 from the source. Four weekly sub-lethal doses of 5.5 6.5 7.5 8.5 9.5 Gy gamma-ray irradiation were delivered at a dose rate of 1 Gy/min as described previously^13^,^21^,^22^. The mice were then removed from the plastic boxes and allowed free access to food and drinking water.

### Histological study

Tissues from bone marrow, testis, thymus, liver, lung, kidney, spleen and colon of euthanized TLR2^−/−^ mice and TLR2^−/−^ mice with different treatments were harvested and subjected to histological assays as described previously. Briefly, tissues were dehydrated in an ascending grade of ethanol, cleared and embedded in paraffin wax. Serial sections of 2–7 microns thick were obtained using a rotatory microtome. The deparaffinized sections were stained routinely using the hematoxylin and eosin (H and E) method. Photomicrographs of the desired sections were obtained using a digital research photographic microscope.

### BrdU labeling

Crypt stem cell survival was determined 5, 14 and 20 days after irradiation by 5-bromo-2′-deoxyuridine (BrdU) incorporation into proliferating crypt cells, using a modification of the microcolony assay as described previously, with a minor change of the secondary antibody. S phase cells were labeled *in vivo* by administering BrdU (i.p., 120 mg/kg) to each mouse 2 hours before euthanasia. Mice were sacrificed and the bone marrow and small intestine were rapidly dissected, fixed in 10% neutral buffered formalin, and embedded in paraffin. Paraffin sections (2–5 μm) were cut perpendicular to the long axis of the intestines. Cells incorporating BrdU were visualized by immunohistochemistry using rat monoclonal anti-BrdU antibodies (Abcam, Cambridge, MA; Cat. # ab6326, dil.1:100) and secondary HRP-conjugated donkey anti-rat IgG antibody (Cell Signaling technology, Beverly, MA).

### Apoptosis assay

Cells from each group were harvested at 48 h post-transfection. Cells were resuspended at a density of 1 × 106 cells/mL in PBS. After double staining with FITC-Annexin V and (PI) using the FITC Annexin V Apoptosis Detection Kit I (BestBio, Shanghai, China), cell were analyzed using FACScan flow cytometer (BD Biosciences) equipped with Cell Quest software (BD Biosciences). Annexin V + PI- cells were counted as apoptosis cells.

### Quantitation of GM-CFU

Granulocyte-macrophage colony-forming units (GM-CFU was assayed in semisolid methylcellulose culture as described previously, with minor revisions. Mononuclear bone marrow cells (BMC) from femora and tibiae of non-irradiated TLR2^−/−^ mice and TLR2^−/−^ mice were pooled. Red blood cells were removed using RBC lysis buffer (eBioscience, San Diego, Ca). BMC were suspended in Iscove’s modified Dulbecco’s medium (IMDM) containing 30% fetal calf serum, 1% bovine serum albumin, 100 uM beta-mercaptoethanol, 10 ng/ml recombinant mouse granulocyte monocyte-colony stimulating factor (mGM-CSF; Biosource Cat.# PMC2016) and 1 ng/ml recombinant IL-3 (mIL-3; ProTech Rocky Hill NJ, USA). One-milliliter aliquots of the BMC suspension were plated in triplicate in 35-mm tissue culture dishes and incubated for 7 days in a humidified incubator at 37 °C with 5% CO_2_. Colonies were counted under a light microscope.

### Statistical analysis

Comparisons between experimental groups and relevant controls (but not survival curves) were performed using a Student’s t-test. Differences in survival of the various groups of mice were assessed using Kaplan-Meier plus Cox Regression Analysis with the SPSS (Statistical Program for Social Sciences) software. The SPSS software generated a P value and Chi-Square value for each analysis; P < 0.05 was considered a statistically significant difference.

## Results

### TLR2^−/−^ Mice were more susceptible to radiation-induced mortality

**Figure 1:**
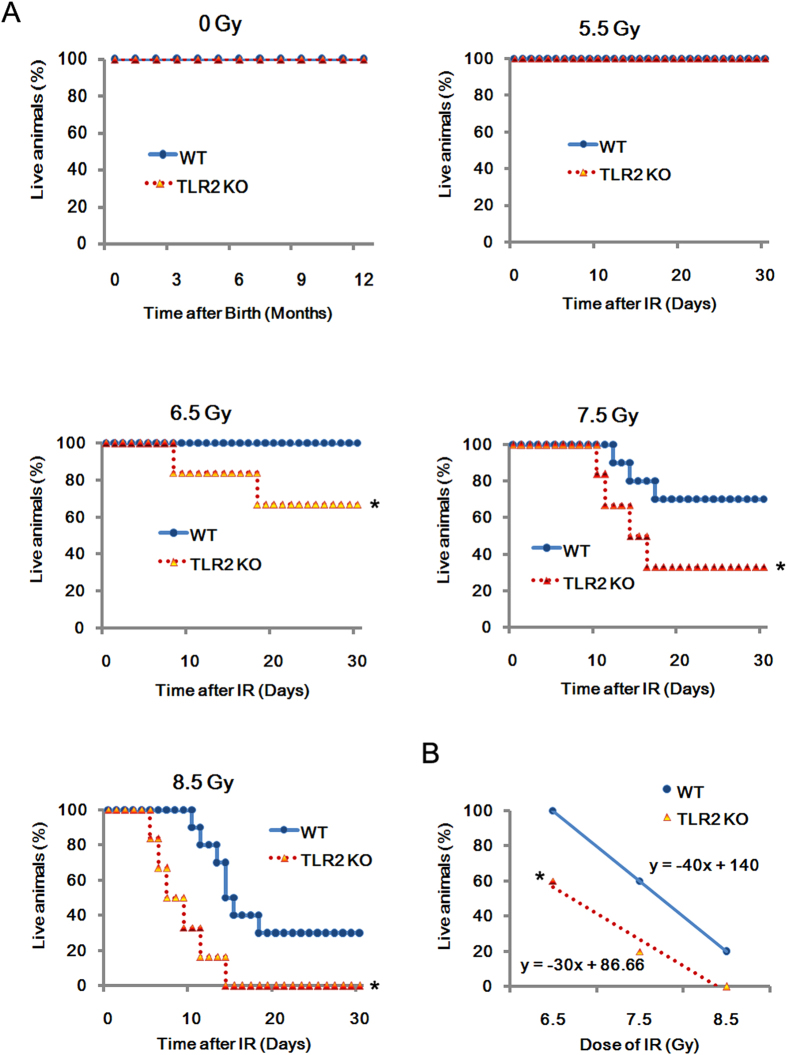
Increased mortality in TLR2^−*/*−^ *mice exposed to IR.* Survival of TLR2^−/−^ and WT mice to month 12 without IR in SPF conditions (*n* = 8). TLR2^−/−^ (*n* = 40) and WT (*n* = 40) mice were randomly divided into four groups and exposed to 5.5, 6.5, 7.5 or 8.5 Gy ^60^Co-γ radiation (dose rate: 1 Gy/min). Survival was monitored until day 30 after IR (**A**) Liner regression analysis of the survival rate for TLR2^−/−^ and WT control mice after IR. The equation and the correlation coefficient for the line are given (**B**) All data are expressed as the mean ± s.d. of three separate experiments. *P < 0.05.

We analyzed the difference in radio-sensitivity between wild type (WT) and TLR2^−/−^ mice under 0, 5.5, 6.5, 7.5, 8.5 Gy of γ-radiation (Fig. 1A). Initially, without radiation, there was no difference between TLR2^−/−^ and WT mice. TLR2^−/−^ mice were able to survive over a year under specific pathogen-free (SPF) conditions. Then, to investigate survivability after exposure to ionizing radiation (IR), age- and sex- matched TLR2^−/−^and WT mice were exposed to 0, 5.5, 6.5, 7.5, 8.5 Gy of γ-radiation. After radiation, the survival rates of TLR2^−/−^ and WT mice were compared. At the dose of 6.5 Gy WT mice had a survival rate of 100%, whereas TLR2^−/−^ mice had a survival rate of only 60% (*p* < 0.05). Consistently, TLR2^−/−^ mice also showed higher mortality than WT mice following exposure to 7.5 or 8.5 Gy radiation. Regression analysis of the survival after radiation exposure confirmed that under the same dose of radiation, there were more deaths in TLR2^−/−^ mice group (Fig. 1B). Taken together, these data, indicated that TLR2^−/−^ mice were more susceptible to radiation-induced mortality.

### Mortality of TLR2^−/−^ mice after radiation was associated with a severe and persistent loss of BMC

**Figure 2:**
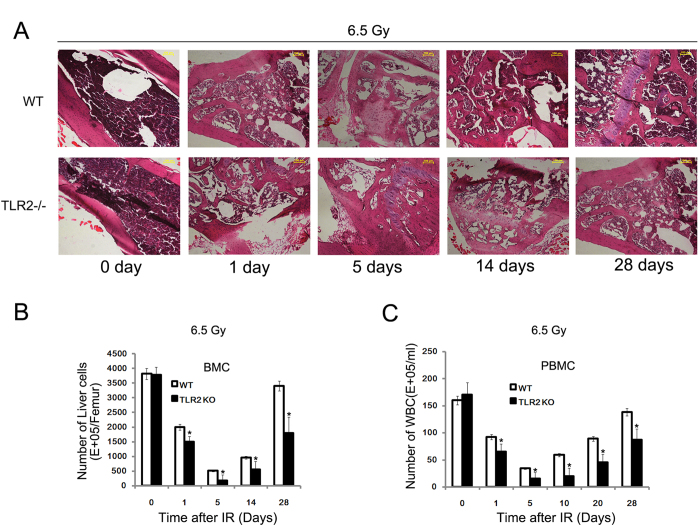
Increased mortality of TLR2^*−/−*^ *mice after IR associated with a severe and persistent loss of BMC.* TLR2^−/−^ and WT mice were irradiated with 6.5 Gy and the femur was collected and stained with H and E at day 0, 1, 5, 14 and 28 post-IR. All images are × 100. The figure shows a typical H and E image of three independent experiments (*n* = 8) (**A**) The number of BMC was counted on day 0, 1, 5, 14 and 28 post-IR (**B**) The number of PBMC was counted at day 0, 1, 5, 14 and 28 post-IR (**C**) *P < 0.05.

To investigate the reason for the increased mortality of TLR2^−/−^ mice, TLR2^−/−^ and WT mice were processed to perform mass biopsy for a histological study of the radiation-induced tissue damage. A 6.5 Gy TBI was found to induce tissue damage in multiple organs of both TLR2^−/−^ and WT mice, but the TLR2^−/−^ mice showed greater injury in bone marrow (Fig. 2A), stomach, spleen, and kidney (Supplementary Figure S1). The histology inspection of these organs suggested that cause of death in TLR2^−/−^ mice may be the severe loss of BMC (Fig. 2A). Hence, to further confirm this result, the number of BMC and peripheral blood mononuclear cell (PBMC) in TLR2^−/−^ were counted after exposure to 6.5 Gy of radiation. We found that 6.5 Gy exposure led more BMC and PBMC loss in TLR2^−/−^ mice. The BM showed the greatest damage and cell loss at day 5 post-radiation, while, by day 28, the bone marrow from WT mice was well repaired and the numbers of cells were partly recovered, this was not the case in TLR2^−/−^ mice (Fig. 2B,C). These data consistently indicate that the main cause of death in TLR2^−/−^ may be a severe and persistent BMC loss after radiation.

### BMC loss in TLR2^−/−^ mice was associated with cell apoptosis and increased oxidative stress

**Figure 3:**
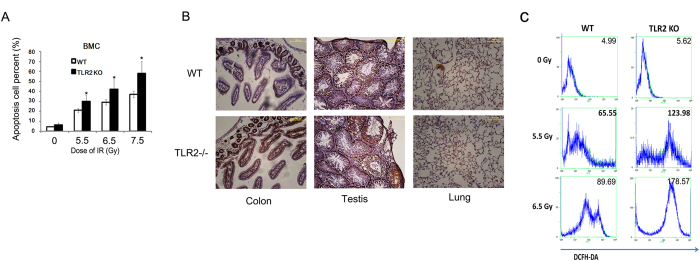
Increased oxidative stress and apoptosis rate of TLR2^*−/−*^ mice after *IR* BMC were prepared 24 h after IR exposure, stained with AnnV-FITV, and PI apoptosis was detected by FACS analysis (**A**) The TLR2^−/−^ and WT mice were irradiated with 6.5 Gy then 1 day later, colon, testis and lung were collected and subjected to TUNEL assay (**B**) The TLR2^−/−^ mice and WT mice were irradiated with 0, 5,5, 6.5 Gy; next, 24 h later, BMC were prepared and analyzed for oxidative stress using the DCFH-DA assay (**C**) All data are expressed as the mean ± s.d. of three separate experiments. *P < 0.05.

To investigate whether the loss of BMC is due to the cell apoptosis, we assayed the cell apoptosis rates of BMC from TLR2^−/−^ and WT mice 24 h after exposure to different doses of radiation. We found that that radiation induced BMC apoptosis in a dose-dependent manner in both TLR2^−/−^ and WT mice, and radiation induced more cell apoptosis in TLR2^−/−^mice (Fig. 3A,). TUNEL assays also indicated there were more cell apoptosis in colon, testis and lung in TLR2^−/−^ mice than in WT mice (Fig. 3B). Besides, DCFH-DA assay showed that there were more oxidative stress in TLR2^−/−^ mice than in WT mice (Fig. 3C) .These data indicated that TLR2 was required for radio-resistance by suppression of apoptosis in many tissues, and especially the BMC.

### BMC from TLR2^−/−^ mice showed impaired proliferation capacity

**Figure 4:**
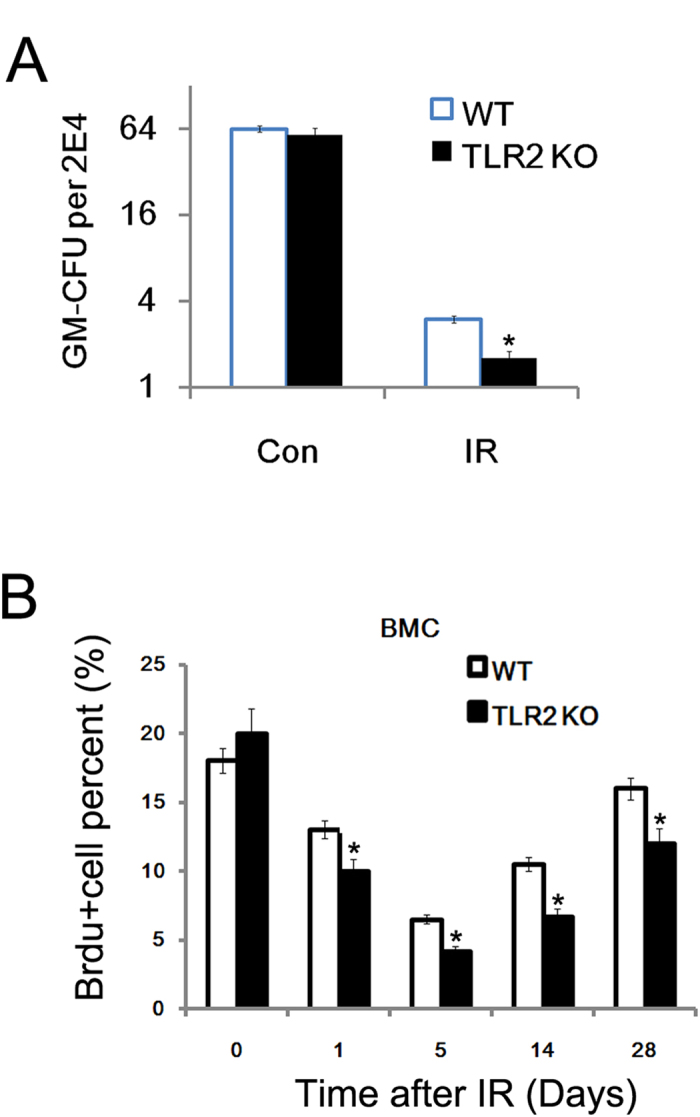
BMC from TLR2^*−/−*^ *mice showed impaired proliferation capacity* Colony-forming units Granulocyte*/*macrophage was quantified in BMC obtained from TLR2^−/−^ and WT mice with or without exposure to IR (6.5 Gy) (**A**) The TLR2^−/−^ and WT mice were exposed to 6.5 Gy, femurs were harvested 5, 14, and 20 days later, and the proliferation of BMC was measured by the BrdU assay. All data are expressed as the mean ± s.d. of three separate experiments. *P < 0.05.

We also performed GM-CFU units and 5-bromo-2′-deoxyuridines (BrdU) to explore why the BM loss was more persistent in TLR2^−/−^ mice. We found that BMC from TLR2^−/−^ mice showed significantly impaired proliferation capacity without IR (Fig. 4A). In addition, the results of the BrdU assay on days 1, 5, 14 and 28 after 5 Gy, revealed that not only the total number of BMC decreased in TLR2^−/−^ mice after TBI, but also irradiated BMC from TLR2^−/−^ mice showed significantly impaired tissues repair capacity when compared to WT mice(Fig. 4B).

### The role of TLR2 agonist Pam_3_CSK_4_ in radio-protection

**Figure 5:**
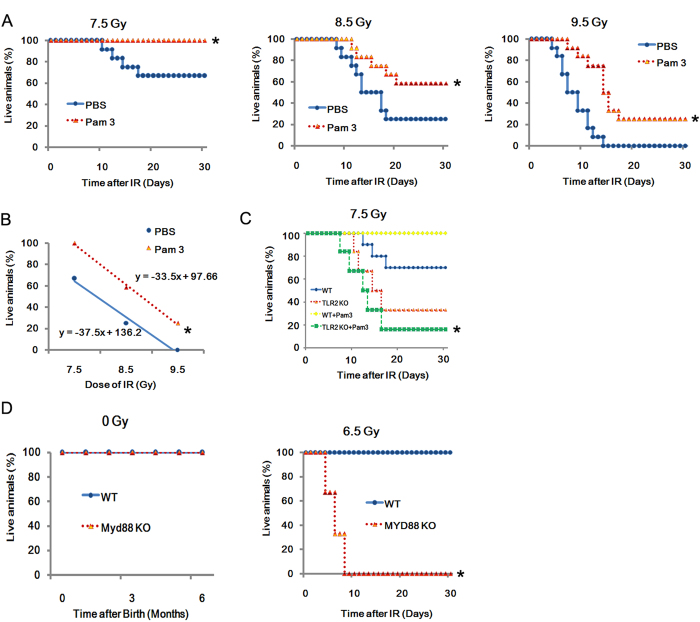
TLR2 induced radio-protection via Myd88 C57BL/10 mice (20 g in weight, *n* = 20 per group) were treated with Pam_3_CSK_4_ (50 ng/ml) and then 24 h later exposed to 7.5, 8.5 and 9.5 Gy TBI as indicated, while control mice received PBS. Survival was recorded (**A**). Linear regression analysis of the survival rate for mice treated with Pam_3_CSK_4_. The equation and the correlation coefficient for the line are given (**B**). Pam_3_CSK_4_ protects C57BL/10 mice from radiation injury. Survival was recorded (**C**). Myd88^−/−^ and WT mice were exposed to 0 and 6.5 IR, and the survival was recorded. All data are expressed as the mean ± s.d. of three separate experiments. *P < 0.05.

Pam_3_CSK_4_ is an agonist of TLR2, and here it was applied to investigate the role of TLR2 in radio-resistance. When given 24 h before radiation exposure, Pam_3_CSK_4_ displayed significant radio-protective effects on C57BL/10 mice exposed to doses of 7.5, 8.5 or 9.5 Gy as shown in Fig. 5A

In addition, the calculated dose-reduction factor (DRF) of Pam_3_CSK_4_ was 1.2 (Pam_3_CSK_4_ *versus* PBS)(Fig. 5B), and when Pam_3_CSK_4_ was given to TLR2^−/−^ mice 24 h before exposure to doses of 7.5 Gy, the results revealed that Pam_3_CSK_4_ did not show a radio-protective effects on TLR2^−/−^ mice (Fig. 5C). Additionally, as the Myd88 dependent pathway is the downstream pathways of TLR2^14^, *in vivo* experiments in Myd88^−/−^ mice were conducted to explored whether Myd88 participate in TLR2-induced radio-protection. The Myd88^−/−^ mice was studies. Myd88^−/−^ mice and WT mice were exposed to 6.5 IR, and the survival was recorded (Fig. 5D). Our data indicated that TLR2 induced radio-protection via Myd88.

## Discussion

In this study, we found that mice deficient in TLR2^−/−^ were more susceptible to IR-induced mortality and morbidity. Mortality in TLR2^−/−^ mice was associated with a severe and persistent loss of BMC. Treatment with the TLR2 agonist Pam_3_CSK_4_ induced radio-resistance. Myd88 may be the critical adaptor for TLR2 mediated radio-protection. Taken together, our data suggested that TLR2 and its agonist Pam_3_CSK_4_ were required for radio-resistance.

Previous study indicated that monocytes incubated with Pam_3_CSK_4_ produced IL-6, IL-8, IL-1β and IL-10^20^, and that IL-6 was a radioprotective cytokine^5^. Indeed, Pam3CSK4 is the potent inducer of IL-12p35 and IL-10 gene expression in murine bone marrow-derived dendritic cells (DCs), as well as in purified oral myeloid DCs. Moreover, sublingual administration of Pam3CSK4, together with the antigen in BALB/c mice sensitized to OVA, dramatically decreases airway hyperresponsiveness as well as OVA-specific T-helper type 2 (Th2) responses in cervical lymph nodes^23^. As our data indicated that it is Myd88 which is involved in the mechanism of the TLR2-induced radio-protection, thus we hypothesized that TLR2-mediated radioprotection is likely to involve multiple mechanisms.

In this and previous study, TLR2^−/−^ or TLR4^−/−^ mice received TBI, and mortality in both mice strain was associated with severe and persistent loss of BMC. Interestingly, exposure of TLR2^−/−^ or TLR4^−/−^ mice to 18-Gy whole thorax radiation has shown that the combined deficiency of these receptors decreases survival time and enhances the development of fibrosis^24^.

Our data indicated the radioprotective role of Myd88. Previous study showed that MyD88 is important for host survival from radiation-induced injury and that in the absence of MyD88, cells accumulated in the lung, which ultimately displayed a fibrotic phenotype by 24 weeks following radiation exposure^25^. These results demonstrate that MyD88 is important for regulating non-infectious inflammatory processes so as to promote healthy tissue regeneration. Besides, there is evidence suggesting MyD88 in regulating hematopoiesis and cell replenishment programs^26^,^27^,^28^.

In conclusion, our data suggest that TLR2 signaling may play a critical role in radio-resistance, at least partly via MyD88.

## Additional Information

**How to cite this article**: Gao, F. *et al.* A critical role of toll-like receptor 2 (TLR2) and its' *in vivo* ligands in radio-resistance. *Sci. Rep.* **5**, 13004; doi: 10.1038/srep13004 (2015).

## Supplementary Material

Supplementary Information
