# Supplementary material for: A critical role of toll-like receptor 2 (TLR2) and its’ in vivo ligands in radio-resistance
Source: Sci Rep. 2015 Aug 13;5:13004. doi: 10.1038/srep13004 (PMC4534783; doi:10.1038/srep13004)
Supplement: Supplementary Information [file srep13004-s1.pdf]

A critical role of toll-like receptor 2 (TLR2) and its' *in vivo* ligands in radio-resistance

Fu Gao[1#], Chaoxiong Zhang[2#], Chuanfeng Zhou[1#], Weimin Sun[3], Xin Liu[4], Pei Zhang[1], Jiaqi Han[1], Linfeng Xian[1], Dongchen Bai[1], Hu Liu[1], Ying Cheng[1], Bailong Li[1], Jianguo Cui[1], Jianming Cai[1*], Cong Liu[1*]

[1]Department of Radiation Medicine, Faculty of Naval Medicine, Second Military Medical University, Shanghai 200433, PR China;

[2]Department of Centre for Disease Prevention and Control, Chengdu Military Region, Chengdu610021, China

[3]National Key Laboratory of Medical Immunology& Institute of Immunology, Second Military Medical University, Shanghai 200433, China

[4]Model Animal Research Center, Nanjing University, Nanjing, People's Republic of China

[#]These authors contributed equally to this paper.

*Correspondence: Jianming Cai & Cong Liu Address: Department of Radiation Medicine, Second Military Medical University; 800, Xiangyin Road 200433, Shanghai; P.R. China Fax: +86-21-81871149.

E-mail: victorliu20102020@163.com & caijianming882003@163.com.

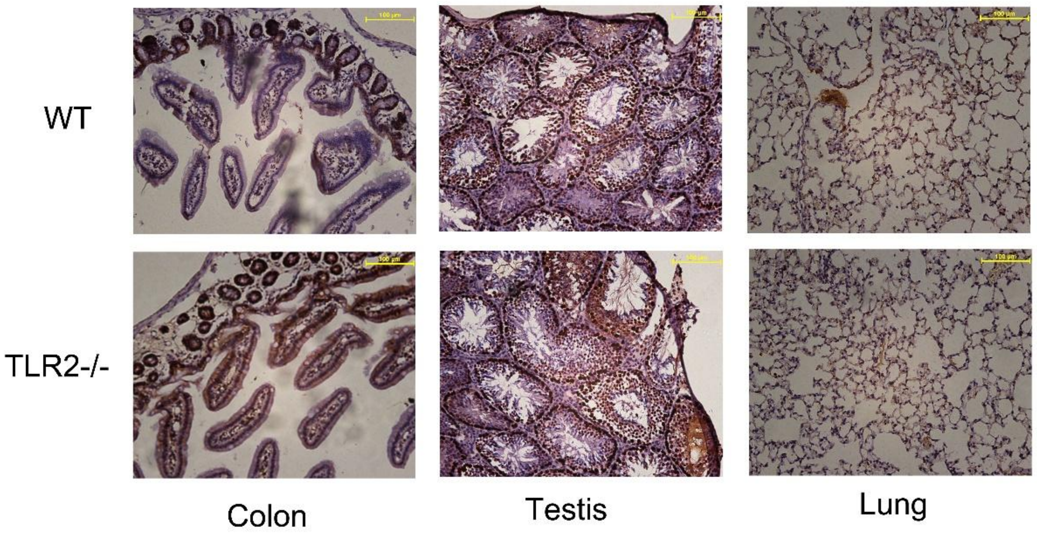

Figure S1

$TLR2^{-/-}$ mice exhibited tissue damage after IR

$TLR2^{-/-}$ and WT mice were irradiated with 6.5 Gy; then, 1 day later, stomach, spleen and kidney tissues were collected and stained to an H and E. The images of the stomach and kidney images are magnified 100×; while spleen is magnified 50×
